# Students' Perceptions of and Experiences With Educational Technology: A Survey

**DOI:** 10.2196/mededu.5135

**Published:** 2016-05-18

**Authors:** Kenneth Royal, Mari-Wells Hedgpeth, Dan McWhorter

**Affiliations:** ^1^College of Veterinary MedicineDepartment of Clinical SciencesNorth Carolina State UniversityRaleigh, NCUnited States; ^2^College of Veterinary MedicineOffice of Academic AffairsNorth Carolina State UniversityRaleigh, NCUnited States; ^3^College of Veterinary MedicineEducational Support ServicesNorth Carolina State UniversityRaleigh, NCUnited States

**Keywords:** medical education, veterinary education, information literacy, experience, attitudes, preferences, technology

## Abstract

**Background:**

It is generally assumed that incoming students in medical education programs will be better equipped for the “digital age” given their younger age and an educational upbringing in which technology was seemingly omnipresent. In particular, many assume that today's medical students are more likely to hold positive attitudes and increased comfortability with technology and possess greater information technology (IT) skills.

**Objective:**

The purpose of this study was to compare responses of incoming veterinary medical students to a series of IT-related questions contained in a common questionnaire over the course of a 10-year period (2005-2015) to discern whether students’ attitudes have improved and uses and comfortability with technology have increased as anticipated.

**Methods:**

A survey measuring attitudes and preferences, computing experience, and technology ownership was administered each year for the past 10 years to incoming veterinary medical students at a large veterinary school in the United States. Students' responses to survey items were compared at 3 data points (2005, 2010, and 2015).

**Results:**

Today's incoming veterinary medical students tend to indicate the same desire to improve skills using spreadsheets and web page design as incoming students from 10 years ago. It seems that despite technological advances and increased exposure to such applications and skills, there remains a challenge for students to “keep up” with the ever evolving technology. Moreover, although students continue to report they are very comfortable with using a computer (and related devices), many use their computers as typewriters or word processors, as opposed to a means for performing more advanced computing functions.

**Conclusions:**

In general, today's medical students are not expert computer users as many assume. Despite an upbringing in a digitized world, many students still lack many basic computing skills.

## Introduction

### Comparing 10 Years of Veterinary Medicine Students' Attitudes Toward, Uses of, and Comfortability With Technology

Strong information technology (IT) and computing skills are essential for students across virtually every medical education program (eg, medicine, pharmacy, dentistry, veterinary, and so forth). Given the increased emphasis of electric medical records and efforts to digitize information for quicker and easier retrieval [1-2], the need has never been more pressing. Furthermore, for medical program graduates to promote their practice, they will need to be relatively savvy with IT for marketing and management or administration purposes [3-4].

At the same time, technology is ever-evolving. As incoming students enter various medical programs throughout the world, there is often an assumption that because these students are typically younger, they will be better equipped for the “digital age.” For example, Facebook was released in 2004; thus, one might assume that virtually every modern medical student would be fluent in web page design. One might assert that today's medical students are more likely to hold positive attitudes and experience increased comfortability with technology, as well as possess greater IT skills [5-6]. The purpose of this study was to compare responses of incoming veterinary medical students to a series of IT-related questions contained in a common questionnaire over the course of a 10-year period (2005-2015) to discern whether students’ attitudes have improved and uses and comfortability with technology have increased as anticipated.

## Methods

### Instrumentation

A technology survey was administered each year for the past 10 years to all incoming freshmen in the Doctor of Veterinary Medicine program at a large veterinary medical school in the United States. The survey consisted of 6 items measuring students' attitudes and preferences, 2 items pertaining to technology ownership and usage, and 11 items measuring computing experience. The survey format was anonymous and voluntary in nature.

### Recruitment and Design

For the present study, we sought to compare students' responses over the last decade by comparing responses at 3 data points: 10 years ago (graduating class of 2009), 5 years ago (graduating class of 2014), and the present year (graduating Class of 2019). The following statistics provide an overview of class sizes and response rates: class of 2009 had 38 of 75 (50.6%) participants; class of 2014 had 51 of 80 (63.8%) participants; and the class of 2019 had 99 of 100 (99.0%) participants. SPSS statistical software was used to perform all data analyses.

## Results

### Demographics

As noted previously, demographic data were not collected for survey respondents. This was due in part to the potential for social desirability bias among subpopulations as the veterinary student demographic largely includes females, which is a national trend. However, demographic data are available for the entire incoming class cohorts during these years. Table 1 provides a breakdown by year according to gender, race or ethnicity, and age.

### Reliability

Cronbach alpha estimates were generated to evaluate internal consistency. Overall reliability was .86 when evaluating all 17 quantitative items. Cronbach alpha estimates for attitude and preference items and computing experience items were .67 and .89, respectively. These values indicate low-moderate to moderate statistical reproducibility [7].

### Attitudes and Preferences

Students were asked to provide their agreement with 6 items measuring attitudes and preferences using a 5-point Likert-type scale (1 = strongly disagree; 5 = strongly agree). Descriptive statistics were produced for each item (see Table 2). Analysis of variance (ANOVA) results indicated that no statistically significant differences (*P*<.05) were discernible when comparing responses to each item based on class year.

**Table 1:** Demographic characteristics on incoming class by graduating year.

Characteristic		2009, N=75, n(%)	2014, N=80, n(%)	2019, N=100, n(%)
Sex				
	Male	15 (20.0)	12 (15.0)	24 (24.0)
	Female	60 (80.0)	68 (85.0)	76 (76.0)
Race or ethnicity				
	White	61 (81.3)	52 (65.0)	71 (71.0)
	Black	7 (9.3)	7 (8.8)	8 (8.0)
	Hispanic	3 (4.0)	3 (3.8)	4 (4.0)
	Other	4 (5.3)	18 (22.5)	16 (16.0)
Age (mean), years		24.4	23.4	24.5

**Table 2:** Responses to attitudes and preferences items.

Item	Class	N	Mean	SD^a^	CI^b^ (95%)
I am confident about my ability to use a computer for general course work	2009	38	4.79	0.577	4.60-4.98
	2014	51	4.92	0.272	4.85-5.00
	2019	98	4.83	0.381	4.75-4.90
I want the opportunity to use computers as much as possible in my course work	2009	38	3.89	1.060	3.55-4.24
	2014	51	3.84	0.925	3.58-4.10
	2019	99	4.01	0.851	3.84-4.18
I want to access course materials online	2009	38	4.58	0.683	4.35-4.80
	2014	51	4.69	0.583	4.52-4.85
	2019	99	4.79	0.500	4.69-4.89
I would be interested in taking some courses designed to use web, video, email, and other technologies so that I can work from home rather than attend class (or attend class less often)	2009	38	3.26	1.349	2.82-3.71
	2014	51	3.10	1.330	2.72-3.47
	2019	99	3.47	1.358	3.20-3.75
I want my instructors to use technology in the classroom for presentations and demonstrations	2009	38	4.42	0.722	4.18-4.66
	2014	51	4.33	0.554	4.18-4.49
	2019	99	4.39	0.682	4.26-4.53
I prefer to answer this type of survey online as opposed to paper	2009	38	4.71	0.565	4.52-4.90
	2014	51	4.63	0.799	4.40-4.85
	2019	98	4.47	0.976	4.27-4.67

^a^SD: standard deviation.

^b^CI: confidence interval.

### Computing Experience

Students were asked to rate their proficiency with respect to 11 items measuring computing experience by using the following scale: 1 = poor; 2 = below average; 3 = average; 4 = good; 5 = excellent. Descriptive statistics were produced for each item (see Table 3). An ANOVA was initially performed, but a Levene's test of homogeneity of variances indicated that 6 of the 11 items possessed significantly different variances (*P*<.05), thus indicating that parametric statistical procedures should not be used. A Kruskal-Wallis nonparametric test was instead performed to investigate potential differences in responses across the class year variable. Because SPSS does not report effect size estimates for Kruskal-Wallis tests, eta square estimates were computed manually using the formula: η^2^= *χ*^2^/ *N* -1, where *χ*^2^ is the chi-square value and *N* is the total number of cases. Results indicate that 3 of the 11 items yielded statistically significant differences. Furthermore, effect size estimates for each item were medium in magnitude, indicating a moderate “practical significance” [8].

### Technology Ownership

Students were asked “what operating system(s) do you run on your computer?” and “what web browser do you primarily use on your computer?” The results are summarized in Table 4.

**Table 3:** Responses to computing experience items.

Item	Class	N	Mean	SD^a^	CI^b^ (95%)	*P*	*η*^2^
Delete, move, rename, or copy files	2009	38	4.24	0.97	3.92-4.56	.001	.08
	2014	50	4.84	0.42	4.72-4.96		
	2019	99	4.67	0.69	4.53-4.80		
Create and manage folders and directories	2009	38	3.97	1.13	3.60-4.34	.011	.05
	2014	51	4.53	0.64	4.35-4.71		
	2019	99	4.57	0.70	4.43-4.71		
Perform general formatting on text (change font sizes, select, copy, move, center, underline, bold, number pages, and so forth)	2009	38	4.68	0.74	4.44-4.93	.946	.00
	2014	51	4.82	0.39	4.72-4.93		
	2019	99	4.76	0.57	4.64-4.87		
Insert and modify figures, images, tables, and so forth	2009	38	4.05	0.99	3.73-4.38	.030	.04
	2014	50	4.34	0.66	4.15-4.53		
	2019	98	4.47	0.76	4.32-4.62		
Perform general data entry (use different numeric formats, change column widths, insert rows or columns, cut or paste values, and so forth)	2009	38	4.13	1.26	3.72-4.54	.587	.01
	2014	51	4.45	0.832	4.22-4.69		
	2019	97	4.38	0.90	4.20-4.56		
Use functions and formulas for common tasks (sum and average ranges of cells, apply simple financial functions, specify absolute and relative cell addresses)	2009	38	3.50	1.29	3.08-3.92	.757	.00
	2014	51	3.75	1.06	3.45-4.04		
	2019	99	3.67	1.06	3.46-3.88		
Create spreadsheet charts (pie charts, bar charts, and so forth)	2009	38	3.71	1.23	3.31-4.11	.327	.01
	2014	51	4.12	0.91	3.86-4.37		
	2019	99	3.90	1.04	3.69-4.11		
Send, receive, copy, and forward email	2009	38	4.76	0.54	4.59, 4.94	.472	.01
	2014	51	4.90	0.30	4.82-4.99		
	2019	99	4.81	0.53	4.70-4.91		
Send and receive attachments	2009	38	4.71	0.57	4.52-4.90	.160	.02
	2014	51	4.90	0.30	4.82-4.99		
	2019	99	4.82	0.50	4.72, 4.92		
Use the Internet for searching for and locating specific information	2009	38	4.58	0.64	4.37-4.79	.109	.02
	2014	51	4.73	0.57	4.57-4.89		
	2019	99	4.78	0.53	4.67-4.88		
Create your own web pages	2009	38	2.29	1.25	1.88-2.70	.467	.01
	2014	51	2.63	1.28	2.27-2.99		
	2019	99	2.46	1.27	2.21-2.72		

^a^SD: standard deviation.

^b^CI: confidence interval.

**Table 4:** Responses to technology ownership and usage items.

Response		2009, N (%)	2014, N (%)	2019, N (%)
Operating system				
	Windows	37 (97.4)	33 (64.7)	49 (49.5)
	Mac	1 (2.6)	13 (25.5)	40 (40.4)
	Dual or Other	0 (0.0)	5 (9.8)	10 (10.1)
Primary browser				
	Internet Explorer	28 (73.7)	12 (23.5)	8 (8.1)
	Mozilla Firefox	6 (15.8)	30 (58.8)	15 (15.2)
	Chrome	0 (0.0)	0 (0.0)	54 (54.5)
	Safari	1 (2.6)	8 (15.7)	22 (22.2)
	Other	3 (7.9)	1 (2.0)	0 (0.0)

### Most Desirable Computer Skill to Learn

Responses to the open-ended item asking “What computer skills would you like to learn?” varied considerably across class years. With respect to the class of 2009, 28 of the 38 participants provided responses and 17 (60.7%) of them indicated interest in web page design, and 15 (53.6%) expressed an interest in increasing familiarity with Microsoft Excel. With respect to the class of 2014, 27 of 51 participants provided responses and 10 (37.0%) of them indicated interest in Excel, 7 (25.9%) were interested in web page design, and 4 (14.8%) indicated interest in becoming more competent with the use of Microsoft PowerPoint. With respect to the class of 2019, 63 of 99 participants provided responses and 30 (47.6%) of them expressed interest in Excel, and 18 (28.6%) of them were interested in web page design.

## Discussion

### Principal Findings

With regard to students' attitudes and preferences about technology, multiple measures over a 10-year period suggest that little has changed as students indicated comparable levels of agreement to each item over time. With respect to computing experience, a similar trend was discernible with exception to items pertaining to file management (eg, deleting, moving, copying), folder management (eg, creating and managing folders and directories), and data or image manipulation (eg, inserting and modifying figures, tables, images, and so forth). Students' perceived competence with these skills has increased over time.

A review of qualitative comments regarding most desirable computer skills to learn reveals an interesting trend. Ten years ago, most students wanted to learn more about web page design and increase their proficiency with Microsoft Excel. Five years ago, interest in Excel and web page design remained the most sought-after skills, but the percentage of students expressing interest in these skills declined considerably over the previous 5 years. Today, students again express a strong interest in Excel and web page design, but the focus seems to be on more advanced applications using each of these platforms. These findings may suggest that despite technological advances and increased exposure to such applications and skills, there remains a challenge for students to “keep up.”

Perhaps one of the most glaring findings of this study was that the vast majority of students indicated that they were very confident about their ability to use a computer for general course work but still lacked proficiency in some rather basic computing skills (eg, working with spreadsheets, basic web design, and so forth). We contend that these findings suggest students primarily use computers as typewriters, as opposed to a means for performing more advanced computing functions. That is, although virtually every student is familiar with computing devices, there is a significant proportion of students who do not possess skills beyond basic word processing, email, and information retrieval.

### Other Considerations

It is undeniable that medical students must possess a minimum level of computing competence to be successful in today's workplace. However, the question is where do students develop these skills? Medical schools are known to have rigid curricula, in which there is little flexibility to make changes. Much has been written about the difficulties of identifying where curricular cuts and changes should be made and the politics surrounding such implementations [9]. Thus, it seems that offering IT training as part of the medical curriculum is a less-than-ideal solution. Furthermore, incoming medical students come from a wide variety of disciplinary backgrounds, making it unlikely that all students have received adequate formal training as part of their undergraduate education. Should medical schools require a demonstration of technological competence before admission? If so, what might these requirements be, especially in light of the fast-paced and evolving nature of technological advances? Some medical schools provide IT information sessions during student orientation, but are these sessions enough? At present, there is little published research indicating the effectiveness of such sessions.

One potential solution is for medical schools to provide “brown bag” luncheons and workshops to facilitate these skills. Such meetings routinely occur in medical schools where students discuss career specialties, future employment opportunities, research opportunities, and so forth. It seems reasonable for medical education programs to include matters of educational technology as part of such meetings. Another possibility is for educational technology groups, or perhaps campus teaching and learning centers, to produce video or web-based tutorials and have Academic Affairs units with medical schools to require students to complete IT-related training sessions at some point during the curriculum. Each of these possibilities would be quite realistic and should result in little faculty resistance, as they would occur outside the classroom and would not interfere with instructional time. Faculty wishing to incorporate advanced technology skills into their courses (eg, data manipulation in Excel, and so forth) might simply direct students to external resources (eg, online tutorials, and so forth) when assigning work, as this will ensure that students devote requisite time and attention to acquiring necessary IT-related skills while generating work products.

There is also an inescapable ethical element involving medical students and technology. Much has been written about students' perceptions, attitudes, and uses of social media and various aspects of professional behaviors [10], but little has been discussed regarding the potential consequences that may result from a lack of competence with computers and various aspects of IT. For example, unscrupulous individuals are constantly searching for security vulnerabilities to exploit users. Phishing scams, sophisticated viruses, and a host of other issues can wreak havoc on one's career, especially in instances in which sensitive information (eg, patient data) are compromised. Furthermore, medical education faculty are not usually the best models for how to use technology; thus, when students see faculty struggle with technology, it could potentially further deter them from taking action to improving their own technological skills. For these reasons, we argue medical schools have a moral obligation to, at the very least, inform students (and faculty) of the potential consequences that may result from a host of security threats and offer some “best practices” advice for safe computing.

Wu et al [11] noted that students in medical programs possess a wide array of devices; thus, it is critical to continually monitor this information each year to better forecast future needs in related areas, such as libraries. Understanding students' attitudes, preferences, and the types of devices they use can help library staff remain flexible and ensure that timely resources are available for students. We also anticipate information from surveys such as the one described in this paper will be of use to some faculty and key administrators (eg, academic deans, research deans, and so forth) as they begin efforts to plan a course, try a new instructional approach (eg, online testing, project-based learning activity, and so forth), create policies pertaining to academic conduct and professional expectations, and so forth.

### Limitations

A potential limitation of this study is that demographic data were not collected from respondents; thus, we are unable to compare responses to various items by key demographic criteria. However, we believe offering anonymity to complete the survey likely improved the accuracy of the findings. More specifically, most student populations in veterinary medical schools are predominantly female; thus, acquiring demographic information may have caused some students from underrepresented groups (eg, males) to respond in a socially desirable manner given it would there would be a greater possibility of identifying these students. Another potential limitation is sample size. Despite respectable response rates, student cohorts across the 10-year period ranged from 75 to 100 students.

### Conclusions

It is generally assumed that incoming students in medical education programs will be better equipped for the “digital age” given their young age and an educational upbringing in which technology was pervasive. Consequently, many assume that today's medical students are more likely to have a positive attitude toward and increased comfortability with technology and possess greater IT skills. We sought to test this assumption by comparing students' responses obtained from an IT-related survey administered to incoming freshmen each year for the past 10 years. Results indicate that today's incoming students express the same deficiency in IT-related skills as students from previous years, suggesting that it is a challenge for students to “keep up” with technological advances. Furthermore, although it is true that students typically report being comfortable with the use of computers (and similar devices), there is evidence to suggest that students primarily use these devices as typewriters, as opposed to a platform for performing advanced operations. We conclude that it is an erroneous assumption that today's students are much more skilled in the area of IT than students from past years. The good news, however, is that modern medical students recognize the need for increased IT-related skills and indicate a desire to learn these skills.

## Abbreviations

IT: information technology
